# Room-temperature magnetic topological Weyl fermion and nodal line semimetal states in half-metallic Heusler Co_2_TiX (X=Si, Ge, or Sn)

**DOI:** 10.1038/srep38839

**Published:** 2016-12-15

**Authors:** Guoqing Chang, Su-Yang Xu, Hao Zheng, Bahadur Singh, Chuang-Han Hsu, Guang Bian, Nasser Alidoust, Ilya Belopolski, Daniel S. Sanchez, Songtian Zhang, Hsin Lin, M. Zahid Hasan

**Affiliations:** 1 Centre for Advanced 2D Materials and Graphene Research Centre National University of Singapore, 6 Science Drive 2, Singapore 117546; 2 Department of Physics, National University of Singapore, 2 Science Drive 3, Singapore 117542; 3 Laboratory for Topological Quantum Matter and Spectroscopy (B7), Department of Physics, Princeton University, Princeton, New Jersey 08544, USA

## Abstract

Topological semimetals (TSMs) including Weyl semimetals and nodal-line semimetals are expected to open the next frontier of condensed matter and materials science. Although the first inversion breaking Weyl semimetal was recently discovered in TaAs, its magnetic counterparts, i.e., the time-reversal breaking Weyl and nodal line semimetals, remain elusive. They are predicted to exhibit exotic properties distinct from the inversion breaking TSMs including TaAs. In this paper, we identify the magnetic topological semimetal states in the ferromagnetic half-metal compounds Co_2_TiX (X = Si, Ge, or Sn) with Curie temperatures higher than 350 K. Our first-principles band structure calculations show that, in the absence of spin-orbit coupling, Co_2_TiX features three topological nodal lines. The inclusion of spin-orbit coupling gives rise to Weyl nodes, whose momentum space locations can be controlled as a function of the magnetization direction. Our results not only open the door for the experimental realization of topological semimetal states in magnetic materials at room temperature, but also suggest potential applications such as unusual anomalous Hall effect in engineered monolayers of the Co_2_TiX compounds at high temperature.

Topological semimetals (TSM) are electronic strong spin-orbit metals/semimetals whose Fermi surfaces arise from crossings between conduction and valence bands, which cannot be avoided due to nontrivial topology^1^,^2^,^3^. Such new states of topological matter have recently attracted worldwide interest because they may realize particles that remain elusive in high energy physics, exhibit quantum anomalies, host new topological surface states such as the Fermi arc and the drumhead surface states, and show exotic transport and spectroscopic behaviors arising from the novel bulk and surface topological band structures^4^,^5^,^6^,^7^,^8^,^9^,^10^,^11^,^12^,^13^,^14^,^15^,^16^. Two of the most exciting proposed TSM states are the Weyl semimetal^6^,^7^,^17^,^18^,^19^,^20^ and the nodal line semimetal states^21^,^22^,^23^,^24^,^25^. While the Fermi surface of a Weyl semimetal consists of isolated 0D points in *k* space, i.e., the Weyl nodes, the Fermi surface of a nodal-line semimetal is a 1D closed loop, i.e., the nodal line winding in 3D momentum space. Although the first inversion breaking Weyl semimetal was recently discovered in TaAs^6^,^7^,^17^, the time-reversal breaking Weyl and nodal line semimetals remain elusive. The time-reversal breaking (magnetic) Weyl and nodal line TSM states are predicted to show exotic properties beyond the inversion-breaking TSMs such as TaAs. Firstly, ferromagnetic materials usually have considerable electron-electron interaction. Hence, a magnetic TSM is a promising platform to study the interplay between the TSM states and electronic correlation, which may potentially lead to new correlated topological phases^4^,^26^,^27^. Secondly, magnetic Weyl semimetals show the anomalous Hall effect^28^,^29^, i.e., Hall-like conductivity without an external magnetic field. When making such a magnetic Weyl semiemtal into 2D (monolayer), the anomalous Hall conductance may be quantized. To date, the quantum anomalous Hall effect has only been observed in magnetically doped topological insulator thin film samples such as Cr_*x*_(Bi_*y*_Sb_1−*y*_)_2−*x*_Te_3_^30^, which required ultra-low (mK) temperature. By contrast, monolayer samples of magnetic Weyl semiemtals, which are natural ferromagnets, may realize the quantum anomalous Hall effect at significantly higher temperature, and therefore make this novel phenomena relevant in actual applications^28^. Thirdly, the superconducting proximity effect of a magnetic Weyl semimetal is predicted to show topological Weyl superconductivity^31^. In such an exotic topological superconductor, the superconducting gap has point nodes, which are Weyl nodes, and the Weyl nodes are connected by Majorana Fermi arcs on the surface.

Despite interest, to date, magnetic topological semimetals remain experimentally elusive. One main difficulty is that ferromagnetic semimetals, regardless of its topological trivial/nontrivial nature, are rare in nature. The existing proposed magnetic TSM materials, such as the pyrochlore iridates and HgCr_2_Se_4_^4^,^28^ have relatively low magnetic transition temperatures that are much lower than the room temperature. This fact not only hinders the experimental confirmation but also makes the predicted TSM states inaccessible in actual applications. Here, we present our identification of the room-temperature topological Weyl and nodal line semimetal states in half-metallic Co_2_TiX.

A half-metal is a type of ferromagnet that acts as a conductor to electrons of one spin, but as an insulator or semiconductor to those of the opposite spin. This half metallicity suggests it to be a promising candidate for the TSM states because both the Weyl semimetal and nodal-line semimetal states require crossings between two singly degenerate (spin polarized) bands. In this paper, we explore the possible existence of the TSM states in the half-metallic full Heusler compounds. The half-metallicity in full Heusler compounds including Co_2_MnSi, Co_2_MnGe, Co_2_FeAl_0.5_Si_0.5_, and Co_2_TiX (X = Si, Ge, or Sn) has been a well-known phenomenon in both theory and experiments^32^,^33^,^34^,^35^,^36^,^37^,^38^,^39^,^40^,^41^,^42^,^43^,^44^. Here, we focus on the Co_2_TiX (X = Si, Ge, or Sn).

Electronic band structures were calculated within the density functional theory (DFT)^45^ framework with the projector augmented wave (PAW) method, using the VASP (Vienna Ab Initio Simulation Package)^46^,^47^. The generalized gradient approximation was used to describe the exchange-correlation effects^48^. We used a kinetic energy cut-off of 500 eV and a 16 × 16 × 16-centered *k*-mesh to sample the primitive bulk Brillouin zone (BZ). In order to compute the bulk band structures, we used the experimental lattice constants^32^, *a* = 5.770 Å, *a* = 5.830 Å, and *a* = 5.997 Å for Co_2_TiSi, Co_2_TiGe and Co_2_TiSn, respectively. The spin-orbit coupling was employed in the electronic structure calculations as implemented in the VASP.

Co_2_TiX crystalizes in a face-centered cubic (FCC) lattice with the space group Fm−3m (Fig. 1(a)). Previous magnetic (SQUID) measurements clearly established the ferromagnetic groundstate in these compounds. The relevant symmetries are the 3 mirror planes, 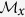 (*k*_*x*_ = 0), 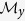 (*k*_*y*_ = 0), 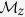 (*k*_*z*_ = 0), and three C4 rotation axes, kx, ky and kz. The Curie temperatures are 380 K for Co_2_TiSi, and Co_2_TiGe and 355 K for Co_2_TiSn. Figure 1(c–e) show the calculated spin-resolved density of states (DOS). We clearly see that, for all three compounds, the band structure is fully gapped at the Fermi level for one spin, i.e., the minority spin, whereas it is metallic for the other spin, i.e., the majority spin. This demonstrates the half-metallic groundstate of Co_2_TiX, consistent with previous theoretical and experimental studies^32^,^33^,^38^,^41^.

Figure 2 shows the first-principles calculated band structure along high-symmetry lines in the absence of spin-orbit coupling. For the band structures of the minority spin (Fig. 2(a–c)), we obtain a minority spin band gap of about 0.5 eV, which is in agreement with previous spin-resolved x-ray absorption spectroscopic measurements^33^. On the other hand, the majority spin band structures (Fig. 2(d–f)) show clear band crossings between the conduction and valence bands along the Γ − *X* and Γ − *K* directions. Furthermore, additional band crossings between the majority and minority spins (highlighted by the black circles in Fig. 2(g–i)) are identified as we overlay the band structures of the two spins.

In order to understand the momentum space configuration of the band crossings without spin-orbit coupling, we calculate the band structure at all *k* points throughout the bulk BZ. Figure 3(a) shows the band crossings in the first BZ. Specifically, we find that the crossings within the majority spin band structure form three nodal lines around the Γ point on the *k*_*x*_ = 0, *k*_*y*_ = 0, and *k*_*z*_ = 0 planes. Figure 3(c) show the energy dispersions along *k*_*x*_, *k*_*y*_, and *k*_*z*_ directions that cut across a *k* point on the *k*_*z*_ = 0 nodal line as noted by the black dot in Fig. 3(a). We see that the two bands disperse linearly away from the crossing point along the radial (*k*_*x*_) direction, while becoming quadratic along the tangential direction. These dispersions confirm the existence of nodal lines. Furthermore, the opposite mirror eigenvalues of the two crossing bands confirm that these nodal rings in the majority spin channels are protected by mirror symmetry. We now consider the band crossings between bands of opposite spins. Since the bands of opposite spins do not hybridize without spin-orbit coupling, the crossings form several 2D closed surfaces, i.e., nodal-surfaces, in the BZ. The situation on the *k*_*z*_ = 0 plane is shown in Fig. 3(d). In addition to the nodal-line (the red lines), four nodal-surfaces also cross this plane inside the four quadrants.

In Fig. 4, we show the band structure after the inclusion of spin-orbit coupling. In the presence of spin-orbit coupling, the symmetry of the system and the electronic structures depend on the magnetization direction. We have calculated the free energy of the system with the magnetization direction along the (001), (110), and (111) directions. Our results show that the difference of the free energy along different magnetization directions is below 0.1 meV, which is beyond the resolution of DFT, suggesting that the system’s magnetization direction can be controlled by an external magnetic field. We present systematic calculation results with the (001) magnetization. With the magnetization along the (001) direction and in the presence of spin-orbit coupling, only the 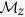 mirror symmetry and the *C*_4*z*_ rotational symmetry are preserved. Hence we expect only the nodal line on the *k*_*z*_ = 0 plane to survive. Indeed, we found this to be the case as shown in Fig. 4(a,b). Near the *k*_*x*_ and *k*_*y*_ axes, the nodal line is formed by bands of the same (majority) spin and is indicted by red color. By contrast, along the *k*_*x*_ = *k*_*y*_ and *k*_*x*_ = −*k*_*y*_ axes (45°), the crossing happens between opposite spins and is denoted by black color. However, as spin is not a good quantum number in the presence of spin-orbit coupling, the above description is approximate. On the other hand, the other two nodal-lines on the *k*_*x*_ = 0 and *k*_*y*_ = 0 planes are gapped out as the respective mirror symmetries are broken by the inclusion of spin-orbit coupling and the (001) magnetization. As a result, we expect Weyl nodes to emerge. Specifically, we find three-types of Weyl nodes, noted as 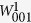, 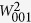, and 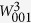 in Fig. 4(a–c), respectively, where the subscript denotes the magnetization direction. The 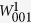 are located on the *k*_*z*_ axis and they are quadratic double Weyl nodes with chiral charge of ±2. The 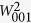, and 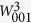 are at arbitrary *k* points in the BZ and are single Weyl nodes with chiral charge of ±1. The energy and momentum space locations of the Weyl nodes are shown in Table 1. We have also calculated the Chern numbers on different *k*_*z*_ planes, which is plotted in the right panel of Fig. 4(c). The variation of the values of Chern numbers as a function of *k*_*z*_ is consistent with the *k*_*z*_ positions of the discovered Weyl nodes. Figure 4(d) shows the energy dispersion away from the 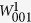 Weyl node. The quadratic 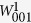 Weyl nodes are protected by the *C*_4*z*_ rotational symmetry. Indeed, we see that the two bands disperse linearly along the *k*_*z*_ direction but quadratically along the *k*_*x*_, *k*_*y*_ directions. Finally, we briefly discuss the band structure with a (110) magnetization as the groundstate. Figure 4(e) shows a comparison between band structures with either a (001) or (110) magnetization direction. It can be seen that the two band structures are very similar, which is consistent with our conclusion that the free energy values with different magnetization directions are quite close to each other. We highlight the area enclosed by the orange box, which is the band structure along the (110) (the *k*_*x*_ − *k*_*y*_ = 0 and *k*_*z*_ = 0) direction. While the band structure with (001) magnetization shows an avoided crossing, i.e., a gap, inside the orange box, the band structure with (110) magnetization shows a band crossing. In this case, the three mirror symmetries (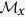, 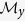 and 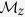) and the three *C*_4_ rotational symmetries (*C*_4*x*_, *C*_4*y*_, and *C*_4*z*_) are broken. The preserved symmetries are the Mirror symmetry 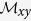 (the mirror plane normal to the (110) direction), the *C*_2_ rotational symmetry along the (110) direction, and the inversion symmetry. Therefore all the nodal rings will gap out. We note that the band crossing along (110) direction is protected as the two bands have the opposite eigenvalues of the *C*_2_ rotational symmetry. We have checked the chiral charge by calculating the Berry flux through a 2D closed surface in *k* space enclosing this crossing point and found that it is indeed a Weyl node. We note that this Weyl node, 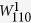, does not exist in the band structure with the (001) magnetization but arise with the (110) magnetization. This fact suggests a novel possibility that the number, the momentum space location, and other properties of the Weyl nodes in the Co_2_TiX system can be engineered by tuning the magnetization direction.

Now we discuss the surface states of Co_2_TiGe in Fig. 5. Figure 5(b) shows the Fermi surface surface states of the Co_2_TiGe(010) surface. The simulation does not take spin-orbit coupling into account. As depicted by orange lines, the projected nodal ring can be clearly resolved on this surface. Moreover, surface state contours starting from the nodal ring could be observed. The energy dispersion along the white line in panel (b) is shown in Fig. 5(c) and further demonstrates the surface states originating from the bulk nodal ring. Thus we are able to identify the topological nature of the surface states. After turning on spin-orbit coupling, no visible difference between the energy contours in panels (b) and (c) can be observed when compared to the spinless calculation, due to the very small strength of the spin-orbit coupling in this material. Next we attempt to distinguish the Fermi arcs surface states of Co_2_TiGe(010) with the magnetization direction along the z axis. The constant energy contours at the energy of Weyl points 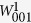 and 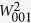 are plotted in Fig. 5(d,e), respectively. Unfortunately, the larger area of the projected bulk pockets covers nearly the entire BZ and hinders the distinction of Fermi arc surface states. This phenomenon can be clearly demonstrated in a E-K dispersion. Figure 5(f) shows the energy dispersion along the red circle in panel (d). The projections of the bulk conduction and valance bands dip into one another and cover the area belong to the Fermi arc surface states.

Although Fermi arc surface states are topologically guaranteed to exist in a Weyl semimetal in the ideal case, the situation in a real material system can become much more complicated. Due to the existence of chiral charge on a Weyl node, a 2D manifold in the BZ, such as a cylinder enclosing the Weyl point, carries a quantized Chern number. As a result, on the surface of an ideal Weyl semimetal, we could resolve Fermi arc surface states as illustrated in Fig. 5(g). However, we note that although quantized Chern numbers are topologically guaranteed, the topological surface states are not always distinguishable in a real material, such as Co_2_TiGe(010). This is because when two bulk pockets of opposite chiral charges are projected onto the same area of the surface, the Fermi arc states are obscured and become extremely difficult to distinguish (Fig. 5(h)). However, we emphasize that the coverage of Fermi arc states by projected bulk pockets does not affect the identification of Weyl nodes and topological node rings in our Hesuler compounds.

We discuss the effect of the onsite Coulomb repulsion *U* to the band structure and the Weyl nodes. We use *U* = 0 in our paper. First, we emphasize that a previous work^32^ on Co_2_TiX that combined both experimental measurements and first-principles calculations showed that the calculated band structure with *U* = 0 actually fits the experimental results better. Therefore, we believe that *U* = 0 better reflects the experimental reality. Nevertheless, we have also calculated the band structure with a finite *U* value. We found that a finite *U* does not have a significant effect on the majority spin band structure. On the other hand, a finite *U* does increase the band gap of the minority spin. Using this as a guideline, we can understand how a finite *U* value affects the Weyl nodes in our calculations. We take the (001) magnetization as an example. As we have discussed in Fig. 4(a), we find three-types of Weyl nodes, noted as 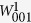, 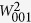, and 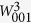. We further note that 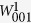 arises from the crossing between two mainly majority spin bands, whereas 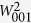 and 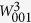 arise from the crossing between one majority spin and one minority spin bands. Therefore, upon the inclusion of a finite *U* value, 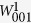 is hardly affected, while 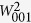 and 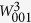 are pushed to higher energies away from the Fermi level. As spin is not a good quantum number in the presence of spin-orbit coupling, this is an approximation. However, as the spin-orbit coupling in Co_2_TiX (X = Si, Ge, or Sn) is not very strong, the mixing between the majority and minority spins is not expected to be signficant.

In conclusion, we have identified the magnetic topological Weyl and nodal line semimetal states in the ferromagnetic half-metal compounds Co_2_TiX (X = Si, Ge, or Sn) with Curie temperatures higher than 350 K. Our results pave the way for realizing topologically protected emergent properties in magnetic semimetals at room temperature, highlighting the potential for electronics and spintronics applications in the Co_2_TiX-based compounds.

## Methods

Electronic structures were calculated within the density functional theory (DFT)^45^ framework with the projector augmented wave (PAW) method, using the VASP^46^,^47^. The generalized gradient approximation was applied to describe the exchange-correlation effects^48^. A kinetic energy cut-off of 500 eV was used for the plane wave basis set and a 16 × 16 × 16-centered k-mesh was used to sample the primitive bulk Brillouin zone. In order to compute the bulk band structures, the experimental lattice constants *a* = 5.830 Å, *a* = 5.997 Å and *a* = 5.770 Å for Co_2_TiGe, Co_2_TiSn and Co_2_TiSi were respectively used. The spin-orbit coupling was employed in the electronic structure calculations as implemented in the VASP. We use *s, p* and *d* orbitals for Co and Ti, and *s* and *p* orbitals for the X (Ge, Si, or Sn) atom to construct the Wannier function^49^. The surface states of a semi-infinite slab were calculated using the iterative Green’s function method from the Wannier function based tight-binding model.

## Additional Information

**How to cite this article**: Chang, G. *et al*. Room-temperature magnetic topological Weyl fermion and nodal line semimetal states in half-metallic Heusler Co_2_TiX (X=Si, Ge, or Sn). *Sci. Rep.* **6**, 38839; doi: 10.1038/srep38839 (2016).

**Publisher's note:** Springer Nature remains neutral with regard to jurisdictional claims in published maps and institutional affiliations.

## Figures and Tables

**Figure 1:**
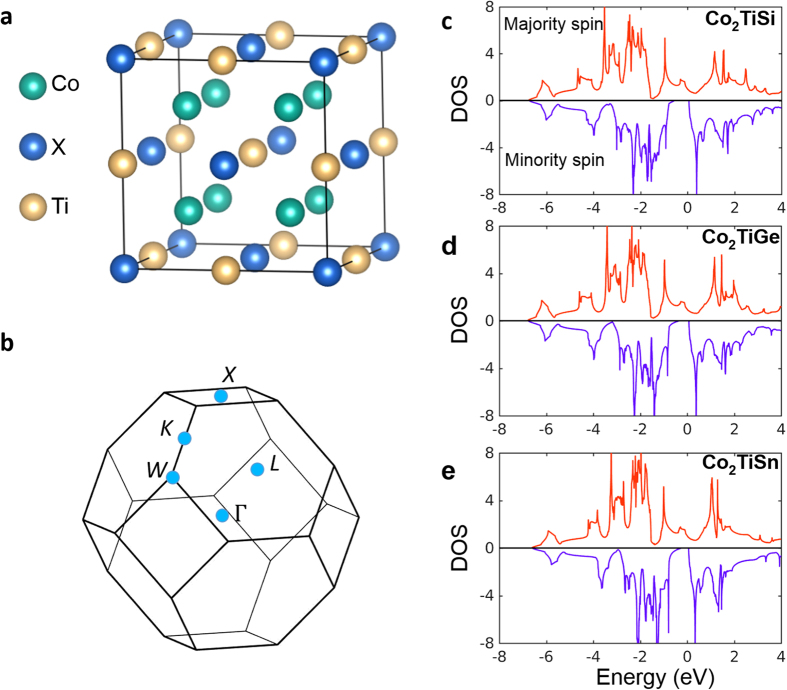
Crystal structure and density of states of the Co_2_TiX compounds. (**a**) The face-centered cubic structure of the Co_2_TiX Heusler compounds. The Co, Ti and X (X = Si, Ge, Sn) atoms are represented by the green, yellow, and blue balls, respectively. (**b**) The first bulk Brillouin zone. High symmetry points are marked. (**c**–**e**) Calculated spin-resolved density of states (DOS) of Co_2_TiX without spin-orbit coupling.

**Figure 2:**
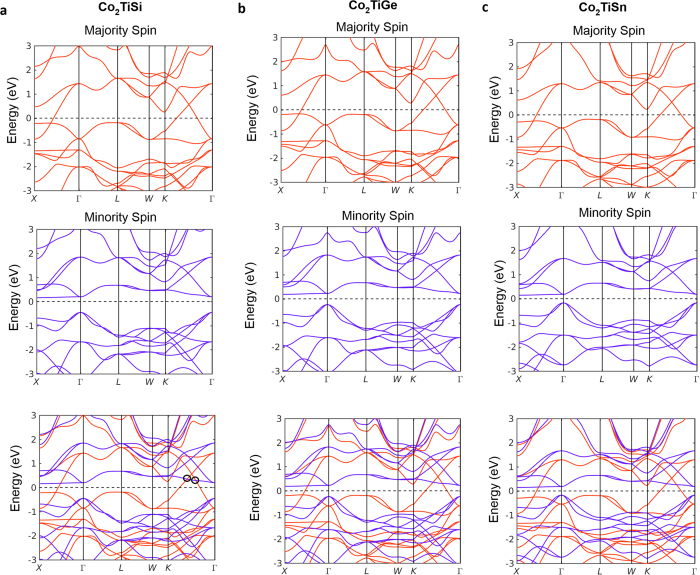
Spin-resolved band structure and ferromagnetic half-metallic ground states in Co_2_TiX. (**a**–**c**) The calculated bulk band structures of the majority spin of the Co_2_TiSi, Co_2_TiGe, and Co_2_TiSn, respectively. (**d**–**f**) Same as panels (a–c) but for the minority spin. (**g**–**i**) The band structures of both spins.

**Figure 3:**
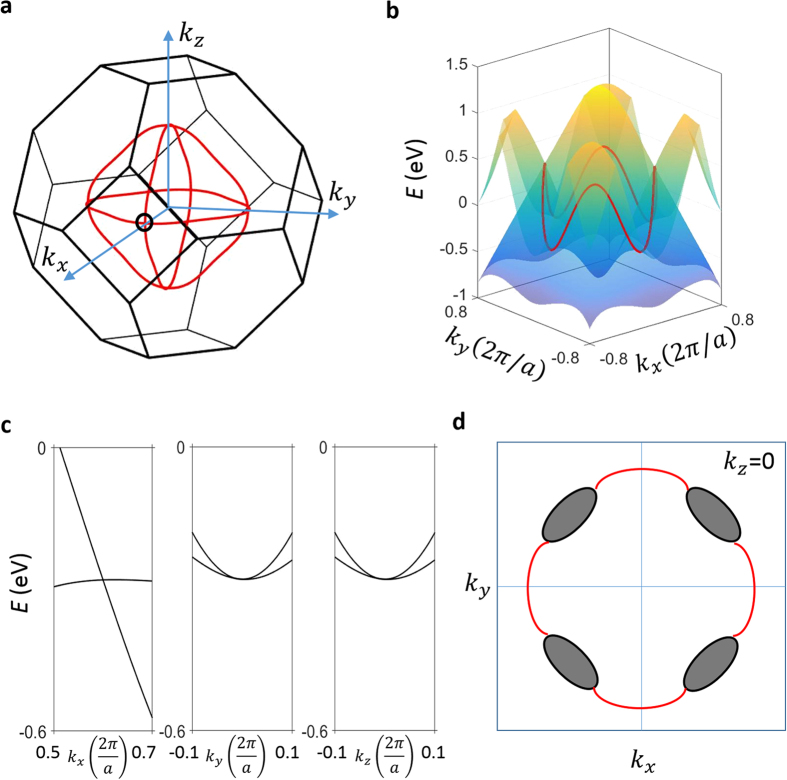
The topological nodal-lines in Co_2_TiX in the absence spin-orbit coupling. (**a**) The nodal-lines in *k*_*x*_-*k*_*y*_-*k*_*z*_ space formed by the band crossings within the majority band structure. (**b**) The band structure in *E*-*k*_*x*_-*k*_*y*_ space containing the nodal-line on the *k*_*z*_ = 0 plane. On *k*_*z*_ = 0 plane, the bulk valence and conduction bands dip into with each other and form a energy-dependent nodal-line (the red line). (**c**) The energy dispersions along *k*_*x*_ (left panel), *k*_*y*_ (middle panel), and *k*_*z*_ (left panel) directions away from the crossing point surrounded by the black circle in panel (**a**). (**d**) Band crossings on the *k*_*z*_ = 0 plane. The red lines show the nodal line formed by the same (majority) spin. The Black ellipses show the cross-sections of the nodal-surfaces formed by opposite spins.

**Figure 4:**
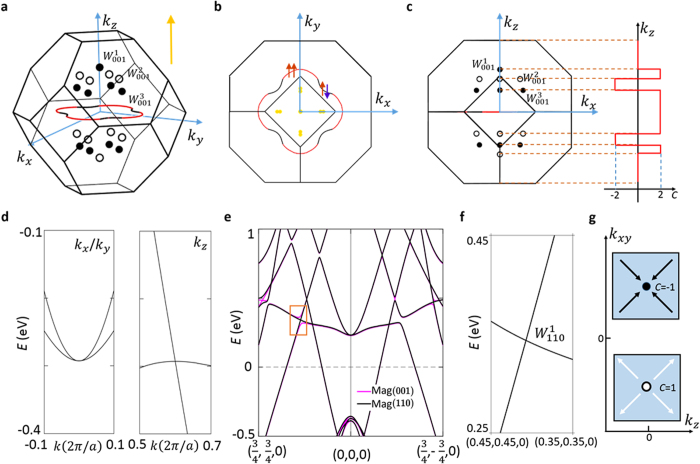
Weyl nodes and nodal-lines in Co_2_TiX in the presence of spin-orbit coupling. (**a**) Band crossings in the first BZ. There are one nodal line on *k*_*z*_ = 0 plane and three types of Weyl nodes (shown by the dots) in the first BZ. The white and black colors denote the chiral charge of the Weyl nodes. (**b**) The projection of the band crossings on the (001) top surface. The projected Weyl nodes are shown by the yellow dots, all of which have a projected chiral charge of 0 as two Weyl nodes of opposite chiralities are projected onto the same point on the (001) surface. The nodal line in panels (**a**,**b**) is shown by the solid lines which are in two colors (red and black). The red segments denote the band crossings formed by two bands of the same (majority) spin whereas the black ones are formed by bands of opposite spins. (**c**) The projection of the band crossings on the (010) side surface (left panel) and the schematic of the Chern number as a function of *k*_*z*_ (right panel). (**d**) The energy dispersions along *k*_*x*_/*k*_*y*_ (left panel) and *k*_*z*_ (right panel) directions of 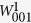 Weyl cone. The quadratic touching of two bands along *k*_*x*_/*k*_*y*_ direction proves that the chiral charge of the Weyl cone is ±2. (**e**) A comparison of the band structures of Co_2_TiX with a (001) or (110) magnetization direction. The number and momentum space locations of the Weyl nodes critically depend on the magnetization directions. (**f**) The zoom-in view of the area indicated by the orange box in panel (**e**). Unlike the case in the (001) magnetization, the band crossing remains intact with the (110) magnetization, and therefore becomes a Weyl node, the 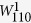. (**g**) A sketch depicting the Berry curvatures of the Weyl nodes in the *k*_*z*_ − *k*_*xy*_ plane. The chiral charge of the Weyl node is denoted.

**Figure 5:**
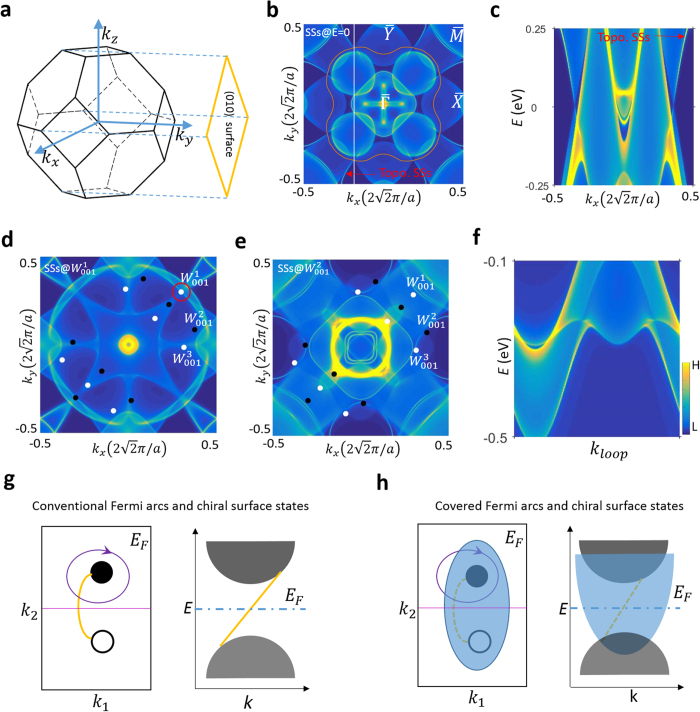
Surface states of Co_2_TiGe(010). (**a**) The first BZ and (010) surface of Co_2_TiGe. (**b**) The Fermi surface of Co_2_TiGe(010), which is derived by a simulation in the absence of SOC. The orange line indicates the projected nodal line on the surface. (**c**) Energy dispersion along the path illustrated by the white line in panel (**b**). (**d**,**e**) Constant energy contours of Co_2_TiX(010) at the energy of of Weyl points 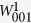, 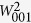, respectively, which are produced by the simulation by considering SOC. The projected Weyl nodes are indicated by black and white balls. The projected bulk band pockets are connected with each other, making the surface states indistinguishable. (**f**) E-k dispersion along the red circles in (**d**). (**g**) A cartoon demonstrating the ideal Weyl cones and Fermi arc, which can both be easily discerned. (**h**) A cartoon showing a realistic case. If a projected bulk band covers the projected Weyl nodes on surface, the Fermi arc merges with the bulk pockets and becomes unobservable. Consequently, the Chern number of the topological 2D manifold can not be identified by the number of chiral surface states.

**Table 1:** Energy and momentum space locations of the Weyl nodes in Co_2_TiGe with a (001) magnetization.

Weyl nodes	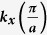	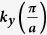	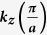	Charge	*E* (eV)
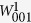	0.00	0.00	0.60	−2	−0.285
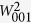	0.00	−0.29	0.46	+1	0.315
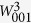	0.00	−0.33	0.30	−1	0.315
